# Decreased apoptotic priming and loss of BCL-2 dependence are functional hallmarks of Richter’s syndrome

**DOI:** 10.1038/s41419-024-06707-5

**Published:** 2024-05-09

**Authors:** Antonella Rigo, Tiziana Vaisitti, Carlo Laudanna, Eleonora Terrabuio, Matilde Micillo, Cristina Frusteri, Beatrice D’Ulivo, Flavia Merigo, Andrea Sbarbati, Kevin Mellert, Peter Möeller, Alessio Montresor, Arianna Di Napoli, Roberto Cirombella, Elena Butturini, Massimo Massaia, Gabriela Constantin, Fabrizio Vinante, Silvia Deaglio, Isacco Ferrarini

**Affiliations:** 1 Cancer Research & Cell Biology Laboratory, Section of Innovation Biomedicine, Department of Engineering for Innovation Medicine, University of Verona, Verona, Italy; 2 Laboratory of Functional Genomics, Department of Medical Sciences, University of Turin, Turin, Italy; 3 Section of General Pathology, Department of Medicine, University of Verona, Verona, Italy; 4 Section of Anatomy and Histology, Department of Neurosciences, Biomedicine and Movement Sciences, University of Verona, Verona, Italy; 5 Institute of Pathology, University Hospital of Ulm, Ulm, Germany; 6 Department of Clinical and Molecular Medicine, Sapienza University, Sant’Andrea University Hospital, Rome, Italy; 7 Department of Neuroscience, Biomedicine and Movement Sciences, Biological Chemistry Section, University of Verona, Verona, Italy; 8 Hematology Unit, Hospital S. Croce e Carle, Cuneo, Italy

**Keywords:** Chronic lymphocytic leukaemia, Chronic lymphocytic leukaemia

## Abstract

Richter’s syndrome (RS) is the transformation of chronic lymphocytic leukemia (CLL) into a high-grade B-cell malignancy. Molecular and functional studies have pointed out that CLL cells are close to the apoptotic threshold and dependent on BCL-2 for survival. However, it remains undefined how evasion from apoptosis evolves during disease transformation. Here, we employed functional and static approaches to compare the regulation of mitochondrial apoptosis in CLL and RS. BH3 profiling of 17 CLL and 9 RS samples demonstrated that RS cells had reduced apoptotic priming and lower BCL-2 dependence than CLL cells. While a subset of RS was dependent on alternative anti-apoptotic proteins and was sensitive to specific BH3 mimetics, other RS cases harbored no specific anti-apoptotic addiction. Transcriptomics of paired CLL/RS samples revealed downregulation of pro-apoptotic sensitizers during disease transformation. Albeit expressed, effector and activator members were less likely to colocalize with mitochondria in RS compared to CLL. Electron microscopy highlighted reduced cristae width in RS mitochondria, a condition further promoting apoptosis resistance. Collectively, our data suggest that RS cells evolve multiple mechanisms that lower the apoptotic priming and shift the anti-apoptotic dependencies away from BCL-2, making direct targeting of mitochondrial apoptosis more challenging after disease transformation.

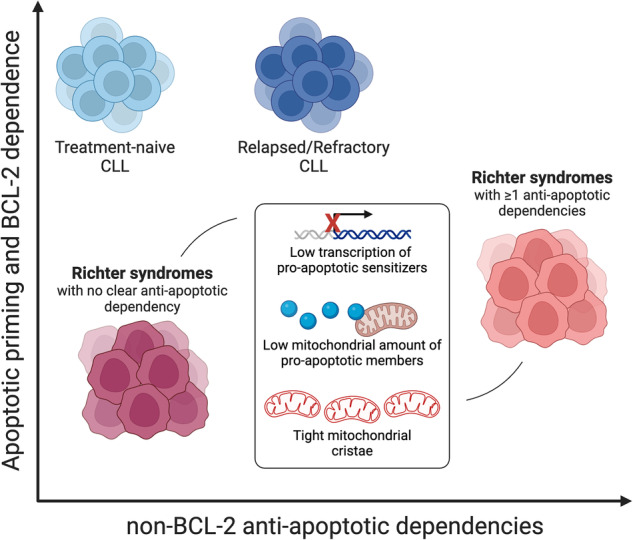

## Introduction

Even in the modern treatment era, 2–10% of patients affected by chronic lymphocytic leukemia (CLL) experience transformation into high-grade histologies, most commonly into diffuse large B-cell lymphoma (DLBCL) [1]. This condition, known as Richter’s syndrome (RS), is characterized by resistance to conventional and targeted agents, with a median overall survival of less than 1 year [1, 2]. Recently, whole-genome sequencing and transcriptomics studies have identified genes and pathways potentially driving RS [3–9]. These include deletions of cell-cycle regulators, reduced expression of chromatin remodelers [3], point mutations of critical transcription factors (TFs) [3, 4, 9], and upregulation of gene sets fostering mitochondrial oxidative metabolism [3]. Despite the contribution of multi-omics analyses, several functional aspects of RS biology have remained unexplored due to the rarity of this disease and the limited availability of live cell-based RS models, two conditions precluding extensive ex vivo investigations.

Among the unexplored aspects of RS, the intrinsic (i.e., mitochondrial) pathway of apoptosis appears of particular interest given its prominent role in the regulation of life/death balance both in homeostasis and during anti-cancer treatments. Intrinsic apoptosis is coordinated at the outer mitochondrial membrane (OMM) by the BCL-2 family of proteins, which are distinguished into anti-apoptotic members (BCL-2, MCL-1, BCL-xL, BFL-1), pro-apoptotic effectors (BAK and BAX), BH3-only activators (BIM, BID, PUMA) and sensitizers (BAD, BMF, HRK, NOXA) [10]. Mechanistically, anti-apoptotic proteins sequester activators and sensitizers, preventing their interaction with BAK and BAX. Following pro-apoptotic stimuli, activators are freed by sensitizers and activate the effector members, which generate pores through the OMM. This favors the leakage of cytochrome c (cyt c) from the intermembrane space and the activation of cytosolic caspases [11]. CLL cells typically express high levels of pro-apoptotic members that are tonically sequestered by BCL-2, thereby having a high apoptotic priming and a functional BCL-2 dependency [12–14].

Here, we used a series of live cell-based RS samples to investigate the apoptotic priming and the anti-apoptotic dependencies of RS. We found that RS cells lose the apoptotic priming and the BCL-2 dependency typical of CLL, highlighting that indolent and aggressive disease phases evade intrinsic apoptosis through divergent mechanisms.

## Results

### RS cells have reduced apoptotic priming

**Fig. 1:**
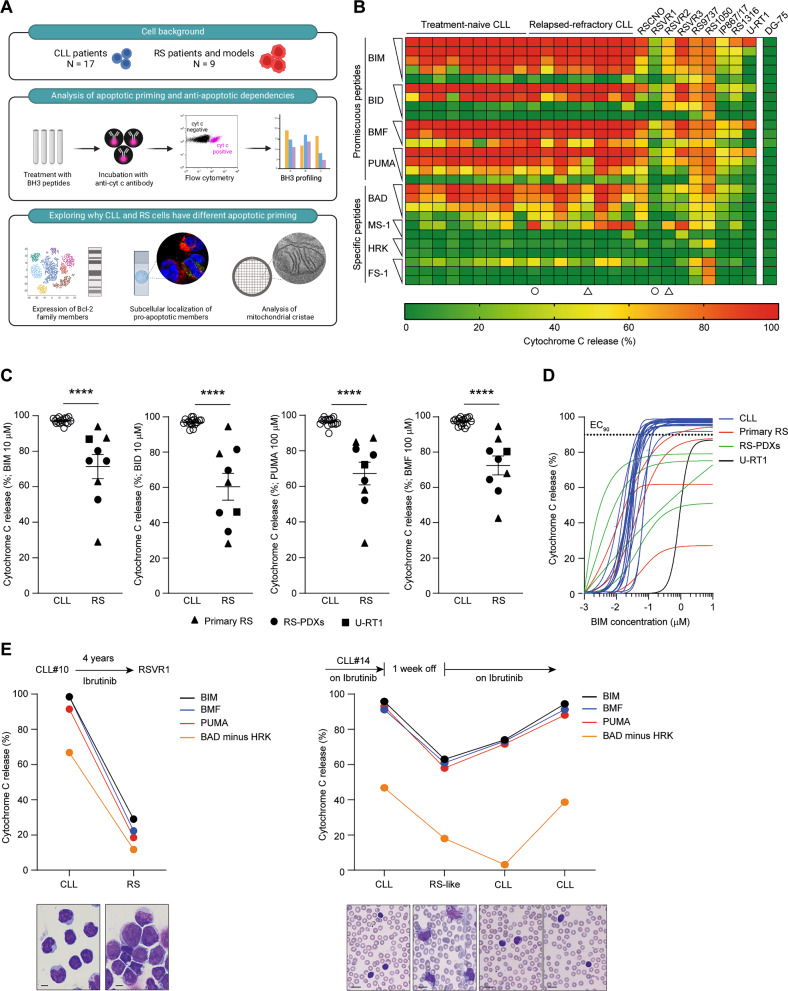
Characterization of RS apoptotic priming. **A** Schematic of study design. Samples from 17 CLL patients and 9 RS patients/models were subjected to intracellular BH3 profiling to derive their apoptotic priming and anti-apoptotic dependencies. Then, a multimodal approach was set up to explore why RS harbored a different functional apoptotic profile as compared to CLL. Particularly, we assessed the expression of BCL-2 family members at transcript and protein level, the subcellular localization of pro-apoptotic effectors and activators, and the width of mitochondrial cristae. **B** Heatmap of the percentage of cyt c loss as quantified by flow cytometry on CLL and RS samples (green, lowest value; red, highest value). Each column is a sample, and each row refers to a BH3-only peptide with which cells were incubated for 60 min. Circles and triangles underneath the heatmap indicate matched CLL/RS samples longitudinally collected from two individual patients. The Burkitt lymphoma cell line DG-75, not expressing BAK and BAX and hence fully resistant to intrinsic apoptosis [54], was used as negative internal control for cyt c release. **C** Percentage of cyt c release for each of the 17 CLL and 9 RS samples upon incubation with the promiscuous peptide (BIM, BID, PUMA, or BMF) indicated on the y axis of each graph. Unpaired Student t test; means ± SEM. ****P < 0.0001. **D** Dose–response curves for BIM peptide. EC_90_ was 0.08 ± 0.09 μM for CLL and not reached for RS samples. **E** BH3 profiling of matched CLL/RS samples. CLL#10 developed DLBCL-type RS (RSVR1) after 4 years of treatment with ibrutinib. CLL#14 developed RS-like transformation (RSVR2) 1 week after abrupt ibrutinib interruption. RSVR2 represents a transient state, as large leukemic B cells reacquired the typical CLL morphology after ibrutinib resumption. All the indicated peptides were used at 10 μM. Images underneath the left graph refer to cytospin of CLL and RS PBMC. Those underneath the right graph refer to peripheral blood smears at different timepoints. Slides were stained with May-Grunwald Giemsa. Original magnification x1000. Scale bar: 10 μm.

To gain insight into the evolution of apoptosis evasion during high-grade transformation, we applied BH3 profiling on 17 CLL (9 treatment-naïve and 8 relapsed/refractory, Supplementary Table 1) and 9 RS samples, and we set up a hypothesis-driven workflow to uncover potential biological determinants of the different anti-apoptotic profile of RS (Fig. 1A, Supplementary Fig. 1). RS cases included 4 primary samples from RS patients in leukemic phase (RSCNO, RSVR1, RSVR2, RSVR3), 4 RS-PDX models (RS9737, RS1050, IP867/17, RS1316), and 1 RS cell line, U-RT1. Among RS primary samples (Supplementary Fig. 2A), RSVR2 was consistent with RS-like transformation due to abrupt ibrutinib interruption [15, 16]. Only RSVR3 patient was treated with venetoclax prior to disease transformation. Four promiscuous pro-apoptotic peptides (BIM, BID, BMF, and PUMA) antagonizing all major anti-apoptotic proteins were applied to permeabilized cells to interrogate the overall apoptotic priming. Overall, RS samples had lower apoptotic priming than CLL cases (Fig. 1B, C). The mean percentage of cyt c release upon incubation with BIM, BID, PUMA and BMF was 97.2, 97.0, 97.5 and 96.4 for CLL versus 71.4, 60.3, 67.1, and 72.5 for RS, respectively (Fig. 1C, *P* < 0.0001 for each peptide). Of note, decreased apoptotic priming was evident in RS but not in relapsed/refractory CLL samples (*P* < 0.001 when comparing relapsed/refractory CLL cases with RS samples), indicating that high-grade transformation and not simply disease relapse or *TP53* mutational status (Supplementary Fig. 3) is required to modulate the apoptotic priming. The median 90% effective maximal concentration (EC_90_) of BIM, a putative biomarker of chemosensitivity [17], was 0.08 ± 0.09 μM for CLL and not reached for all RS cases with the exception of RSVR3 (EC_90_: 0.73 μM; Fig. 1D), consistent with the presence of highly unprimed cell subpopulations within the RS tumor bulk. Notably, matched CLL-RS samples longitudinally collected from two individual patients (CLL#10/RSVR1 and CLL#14/RSVR2) clearly confirmed the loss of apoptotic priming during disease transformation (Fig. 1E). In the RS-like sample RSVR2, the apoptotic priming was reacquired after ibrutinib resumption and concomitantly with the reappearance of the small-cell morphology at the peripheral blood smear (Fig. 1E).

### RS cells show loss of BCL-2 dependence

**Fig. 2:**
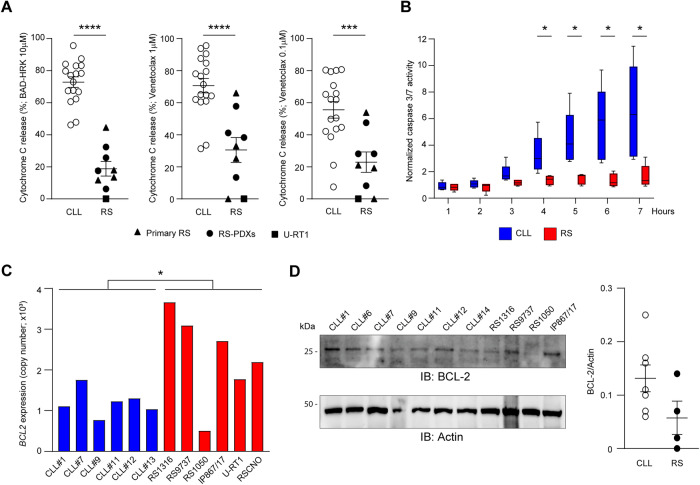
Reduced functional dependence upon BCL-2 in RS cells. **A** Percentage of cyt c release for each of the 17 CLL and 9 RS samples upon incubation with selective peptides (BAD, HRK) or the BCL-2 antagonist venetoclax at the indicated concentrations. As regards peptides, BCL-2 dependence was derived by subtracting the effect of HRK (specific for BCL-xL) from that of BAD (interacting with both BCL-2 and BCL-xL). Unpaired Student t test; means ± SEM. ****P < 0.0001; ***P < .001; **P < 0.01. **B** Real-time monitoring of caspase 3/7 activation upon incubation of 5 CLL and 5 RS samples (4 RS-PDX and RSVR1) with 50 nM venetoclax for up to 7 hours. Whiskers: min to max value. Unpaired Student t test. *P < 0.05. **C** qPCR analysis of *BCL2* in the indicated RS and CLL samples. Unpaired Student t test. *P < 0.05. **D** Western blot analysis for BCL-2 in the indicated CLL and RS samples. The dot plot represents intensity of proteins bands in CLL (open circles) and RS (solid circles). Band intensities were measured using Image Lab and normalized on Actin. Data are reported as mean ± SEM.

To interrogate functional anti-apoptotic dependencies, we incubated CLL and RS cells with an array of BH3 peptides antagonizing specific anti-apoptotic proteins (Fig. 1B, Supplementary Fig. 1A, B). We found that RS cases were less BCL-2 dependent than CLL samples. The mean cyt c release to BAD *minus* HRK peptide, a metric for BCL-2 dependence [18], was 72.8% for CLL versus 18.8% for RS (Fig. 2A, *P* < 0.0001). As with the overall priming, relapsed/refractory status or *TP53* mutations (Supplementary Fig. 3) were not sufficient to drive the loss of BCL-2 dependence, which occurred only in transformed disease (*P* < 0.0001 when comparing relapsed/refractory CLL with RS). Incubation with the small molecule BCL-2 antagonist venetoclax, used the same way as for BH3 peptides, produced similar results with decreased cyt c release in RS (Fig. 2A). Kinetic analysis of caspase 3/7 activation following venetoclax treatment demonstrated induction of apoptosis in CLL cells as soon as 4 h after venetoclax addition. In contrast, RS cells proved insensitive to venetoclax within a 7-hour time frame (Fig. 2B), further indicating their inferior vulnerability to BCL-2 antagonism. Apart from the RS1050 model, which expressed the lowest level of *BCL2* transcript with no detectable protein, all the other tested RS samples showed some degree of BCL-2 expression that was in some case even higher than what observed in CLL (Fig. 2C, D). This indicates that functional BCL-2 dependence in CLL and RS cannot be predicted solely on the basis of BCL-2 expression.

### A subset of RS upregulates alternative anti-apoptotic dependencies

**Fig. 3:**
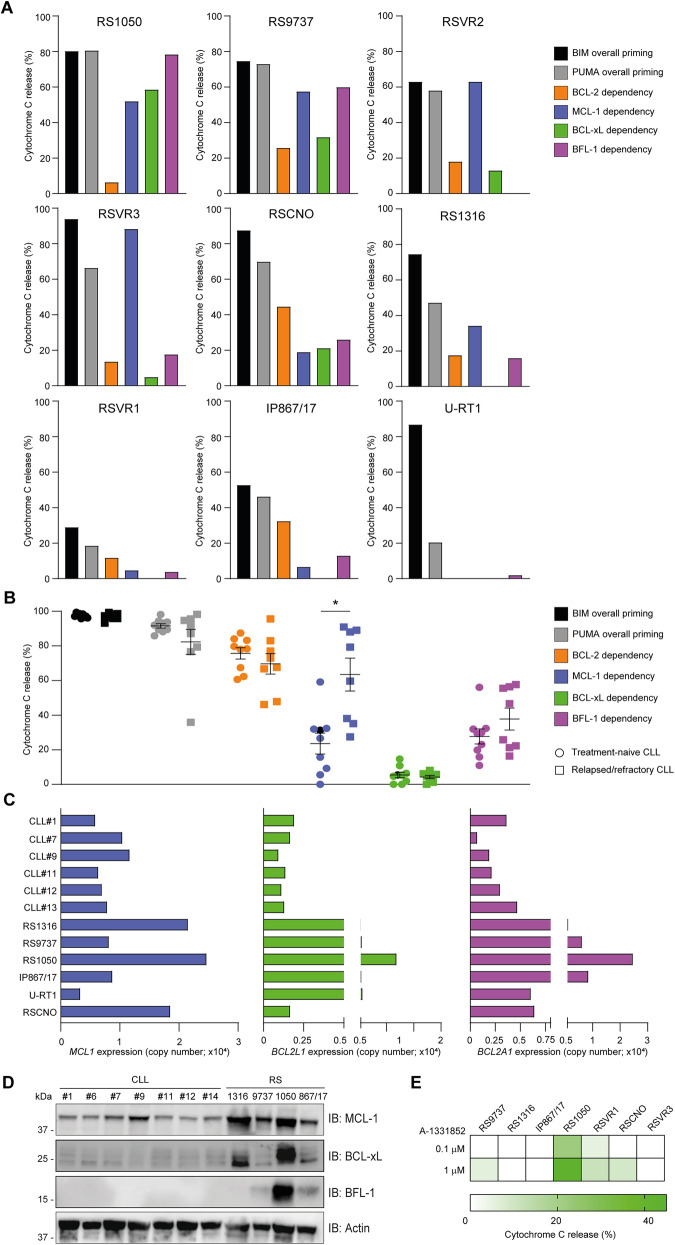
Upregulation of alternative anti-apoptotic dependencies in a subset of RS. BH3 profiling of the indicated RS (**A**) and CLL (**B**) samples. BCL-2 dependence was derived by subtracting the effect of HRK (specific for BCL-xL) from that of BAD. MCL-1, BCL-xL, and BFL-1 dependencies were derived by using MS-1, HRK, and FS-1 peptides, respectively, at the concentration of 10 μM. BIM and PUMA were used at the concentration of 10 μM as well. Unpaired Student *t*-test. *P < 0.05. **C** qPCR analysis of *MCL1*, *BCL2L1* (encoding BCL-xL), and *BCL2A1* (encoding BFL-1) in the indicated CLL and RS samples. **D** Western blot analysis for MCL-1, BCL-xL, and BFL-1 in CLL and RS samples. **E** BH3 profiling of the indicated RS samples after incubation with the specific BCL-xL antagonist A-1331852. *n* = 3.

Having identified a reduced BCL-2 dependence in Richter’s cases, we wondered if some of them upregulated alternative anti-apoptotic dependencies. Out of 9 RS samples, five upregulated alternative dependencies, also showing some degree of co-dependencies (Fig. 3A). Particularly, RS1050 was co-dependent on MCL-1, BCL-xL, and BFL-1, RS9737 was co-dependent on MCL-1 and BFL-1, whereas RSVR3, the RS-like RSVR2 and RS1316 were mainly MCL-1 dependent. The RSCNO case was the only one preserving both a relatively high apoptotic priming and some degree of BCL-2 dependence. The remaining samples displayed more marked loss of PUMA-triggered apoptotic priming and did not show any specific anti-apoptotic dependency (Fig. 3A). Although CLL samples were invariably characterized by high BCL-2 dependence, they acquired co-dependence upon MCL-1 in the relapsed/refractory setting (Fig. 3B). mRNA expression of the anti-apoptotic genes alternative to BCL-2 was partially consistent with the functional data, with RS1050 showing the highest levels for *MCL1*, *BCL2L1* (encoding BCL-xL) and *BCL2A1* (encoding BFL-1) (Fig. 3C). Although there was a trend towards a higher expression of these transcripts in RS, the comparison with CLL was not statistically significant. Western blot analysis confirmed that RS1050 displayed the highest expression of BCL-xL and BFL-1 even at the protein level (Fig. 3D). In addition, we performed BH3 profiling using the selective BCL-xL antagonist A-1331852, and found that RS1050 was the only one showing up to 42.3% of cyt c release in response to this agent (Fig. 3E), indicating that a subset of RS might be targeted by BH3 mimetics that antagonize alternative anti-apoptotic proteins.

### Antagonism of non-BCL-2 anti-apoptotic dependencies and inhibition of proximal signaling pathways are possible ways to trigger apoptosis in RS

**Fig. 4:**
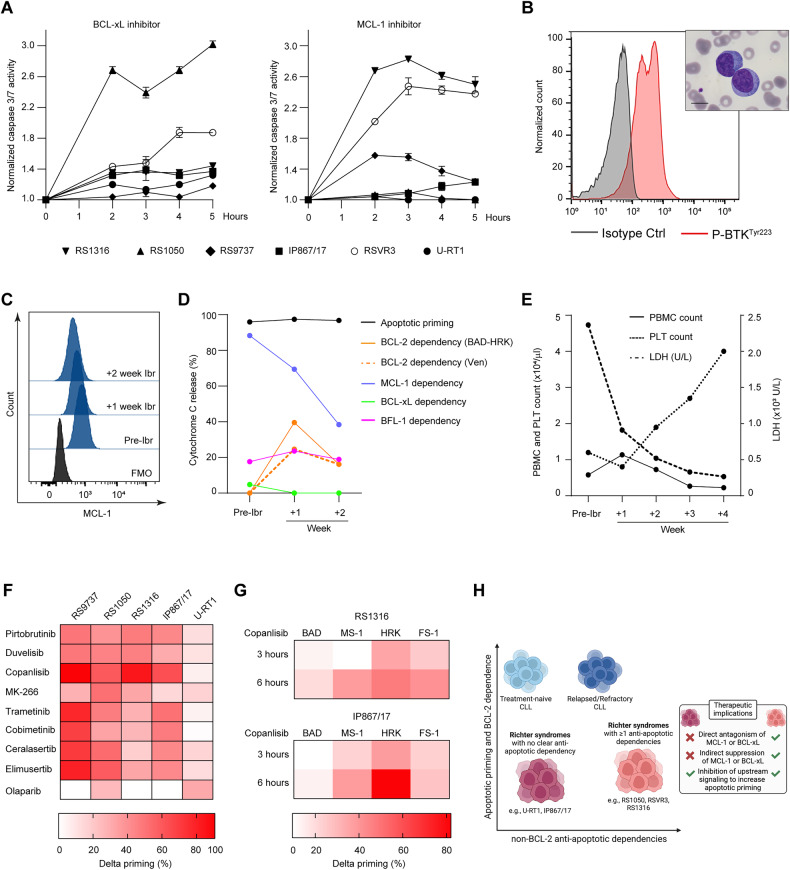
Antagonism of alternative anti-apoptotic dependencies and inhibition of proximal signaling as possible ways to trigger apoptosis in RS. **A** Real-time monitoring of caspase 3/7 activation upon incubation of the indicated RS samples with 50 nM A-1331852 (BCL-xL inhibitor, left panel) or 5 μM S63845 (MCL-1 inhibitor, right panel). As indicated on the y axis, results are normalized on DMSO control for each specific timepoint. *n* = 3. Data are reported as mean ± SEM. **B** Histograms of fluorescence of pBTK^Tyr223^ in leukemic RSVR3 cells. Upper-right inset is the peripheral blood smear of RSVR3 patient showing two typical RS cells characterized by large size, basophilic cytoplasm and one or more nucleoli. Magnification: x1000. Scale bar: 10 μm. **C** Histograms of fluorescence of MCL-1 in leukemic RSVR3 cells before and after ibrutinib (Ibr) start. FMO: fluorescence minus one. **D** BH3 profiling of RSVR3 before and after ibrutinib start. Apoptotic priming was derived by using 10 μM BIM. BCL-2 dependence was derived by subtracting the effect of HRK (specific for BCL-xL) from that of BAD, or by applying 0.1 μM venetoclax. MCL-1, BCL-xL, and BFL-1 dependencies were derived by using MS-1, HRK, and FS-1 peptides, respectively, at the concentration of 10 μM. **E** PBMC and platelet (PLT) count (left y axis), and LDH values (U/L; right y axis) in RSVR3 before and after ibrutinib initiation. **F** Dynamic BH3 profiling. RS samples were treated with the indicated drugs or DMSO for 16 h. Afterwards, they were incubated with BIM peptide. The heatmap shows the delta priming percentage values, corresponding to the cyt c loss of drug-treated cells *minus* cyt c loss of DMSO-treated cells. The higher the delta priming, the more effective the drug is in increasing the apoptotic priming. **G** Extended dynamic BH3 profiling of RS1316 and IP867/17. Cells were treated with 1 μM copanlisib for 3 and 6 h. Then they were subjected to BH3 profiling using the indicated peptides at the concentration of 1 μM. Heatmaps show that in RS1316 cells copanlisib treatment increased the MCL-1, BCL-xL, and BFL-1 dependencies for up to 38%, 47 and 40% at 6 h, respectively. In IP867/17, copanlisib increased MCL-1 and BCL-xL dependencies for up to 38 and 81% at 6 h, respectively. **H** Schematic of CLL and RS apoptotic profiles. CLL are characterized by high apoptotic priming and high BCL-2 dependency, but relapsed/refractory cases can acquire additional, non BCL-2, anti-apoptotic dependencies. In contrast, RS cases are characterized by lower apoptotic priming and BCL-2 dependency than CLL. Based on their BH3 profiling and sensitivity to BCL-xL and MCL-1 inhibitors, RS cases can be further distinguished into those with no clear anti-apoptotic dependency and those with at least one anti-apoptotic dependency. While in the first subset only the targeting of upstream pro-survival pathways can be exploited to increase the apoptotic priming or expose specific anti-apoptotic dependencies, in the latter direct or indirect pharmacological inhibition of the participant pro-survival member can be devised as effective therapeutic approach.

As BH3 profiling revealed that a subgroup of RS harbors non-BCL-2 anti-apoptotic dependencies, we tested whether specific BCL-xL and MCL-1 inhibitors could trigger apoptosis in intact RS cells. RS samples were incubated with A-1331852 or S63845 (a selective MCL-1 inhibitor) and caspase 3/7 activation was monitored over time. In accordance with BH3 profiling, A-1331852 was effective only in RS1050, while S63845 was mostly effective in RS1316 and RSVR3 (Fig. 4A), further suggesting that direct antagonism of BCL-xL and MCL-1 could rapidly promote apoptosis in disease models with such specific anti-apoptotic dependencies. Because the transcription of pro-survival genes alternative to *BCL2* is often driven by upstream oncogenic signaling [19–22], we also reasoned that rational inhibition of key pathways could extinguish non-BCL-2 anti-apoptotic dependencies. RSVR3 patient, a chemo-refractory RS (Supplementary Fig. 2) characterized by the highest apoptotic priming among RS, high MCL-1 dependency (Figs. 3A, 4A) and active BTK signaling (Fig. 4B), was treated with ibrutinib. Two weeks after ibrutinib initiation, MCL-1 expression was reduced in leukemic RS cells (Fig. 4C). Likewise, MCL-1 dependency decreased from 90.8% to 39.9%, with only transient increase of BCL-2 dependency (Fig. 4D). While on ibrutinib, the apoptotic priming remained high and no pro-survival proteins were effectively preventing apoptosis. Such decreased anti-apoptotic protection paralleled with a clear-cut reduction of lactate dehydrogenase (LDH), slow disappearance of RS cells from peripheral blood, and improved bone marrow function (Fig. 4E). To investigate whether inhibition of key signaling pathways could increase the apoptotic priming of RS samples, including those with no anti-apoptotic dependency, we incubated RS-PDXs and U-RT1 cell line with clinical-grade pathway inhibitors, chosen for their putative anti-RS activity based on previous pre-clinical studies [23–25]. We found that pathway inhibitors improved the apoptotic priming in the majority of RS models (Fig. 4F). Copanlisib, a PI3Kαδ inhibitor, was one of the most active compounds in our screen and was able to augment the apoptotic priming even in IP867/17 (Fig. 4F), which had no clear-cut anti-apoptotic dependency at baseline (Fig. 3A). In addition to increasing the overall priming, short term copanlisib treatment exposed multiple anti-apoptotic dependencies (Fig. 4G) potentially exploitable for selective targeting or combinatorial strategies. Collectively, these data suggest that while RS cases harboring non-BCL-2 anti-apoptotic dependencies could be targeted by direct or indirect suppression of the participant anti-apoptotic member, those with no clear anti-apoptotic dependency could be effectively primed (and re-sensitized to apoptosis targeting) by acting on more proximal signaling pathways (Fig. 4H).

### Selected pro-apoptotic sensitizers are transcriptionally downregulated in RS

**Fig. 5:**
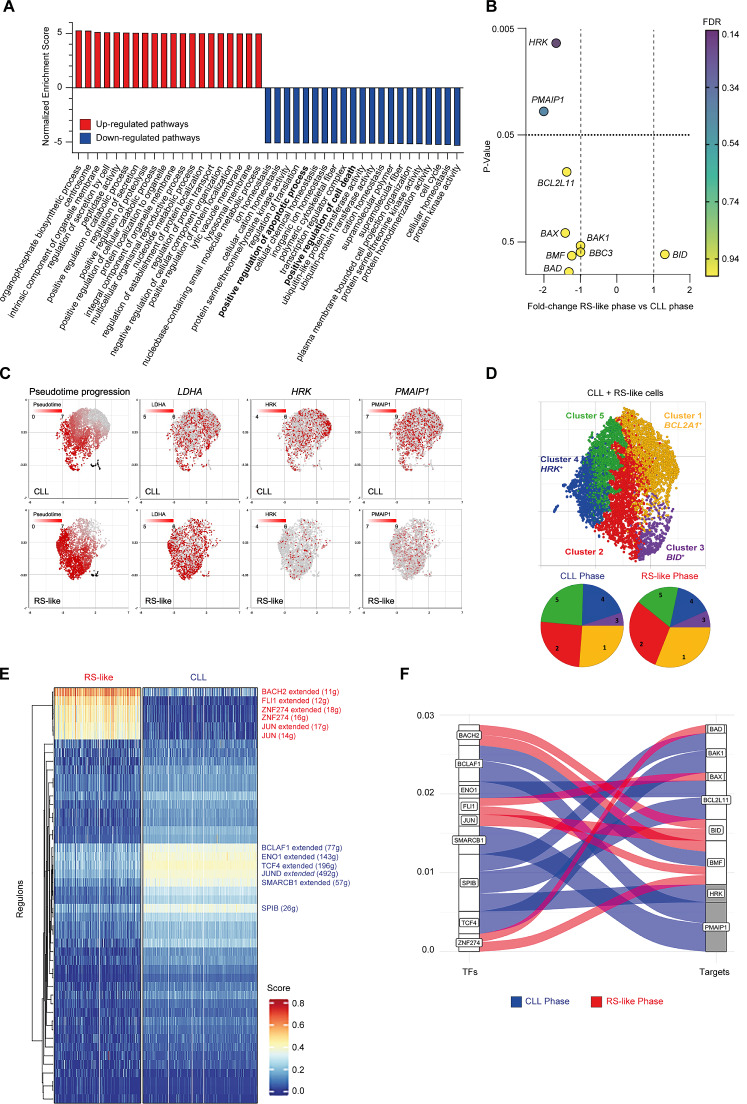
Single-cell transcriptomics of matched CLL/RS samples. **A** Gene set enrichment analysis (GSEA) of the top 20 up- and downmodulated pathways in the RS-like versus CLL phase. Only pathways with >250 genes were considered. The normalized enriched score represents the maximum deviation from zero. *P* < 0.001, FDR < 0.001. **B** Fold change and statistical significance of the pro-apoptotic BCL-2 family genes in the RS-like versus CLL phase. FDR: false discovery rate. **C** Cell fate trajectory analysis showing the pseudotime progression of B cells and the expression of *LDH*, *HRK* and *PMAIP1* along with pseudotime progression. Upper panels: CLL phase. Lower panels: RS-like phase. **D** *Upper panel*. Unsupervised clustering of B cells (CLL + RS-like) showing 5 different clusters with the indicated BCL-2 family biomarkers. *Lower panel*. Cluster distribution in the CLL and RS-like phase. **E** Heatmap illustrating the regulon expression pattern of RS-like and CLL phase after applying SCENIC R-based package to our dataset. Differentially expressed regulons are written on the right side of the heatmap. **F** Alluvial plot showing the BCL-2 family genes (Targets, on the right) that are controlled by the transcription factors (TFs, on the left) identified by gene regulatory network analysis. Blue and red lines refer to TFs enriched in CLL and RS-like phase, respectively. Grey-highlighted BCL-2 family genes (*HRK* and *PMAIP1*) are those differentially expressed in the two disease phases (downregulated in RS-like cells).

To investigate whether decreased apoptotic priming could be driven by transcriptional modulation of pro-apoptotic members, we performed single-cell RNA sequencing on CLL#14 (CLL phase)/RSVR2 (RS-like phase) longitudinal samples. A total of 7762 cells were analyzed, 6271 were B-cells according to Garnett Classification (Supplementary Fig. 4A). Gene set enrichment analysis (GSEA) pathway enrichment analysis showed “positive regulation of apoptotic process” and “positive regulation of cell death” among the top-20 downregulated pathways in the RS-like phase. Conversely, pathways related to structural components of intracellular organelles and regulation of metabolic processes were strongly upregulated (Fig. 5A). Among pro-apoptotic genes of the BCL-2 family, the sensitizers *HRK* and *PMAIP1* (encoding NOXA) were the only ones significantly downmodulated in the RS-like phase. By contrast, none of the pro-apoptotic effectors and activators were modulated during large-cell transformation (Fig. 5B). Cell fate trajectory analysis confirmed the acquisition of a *LDHA*^high^*/HRK*^low^*/PMAIP1*^low^ state along pseudotime progression (Fig. 5C), further highlighting the emergence of proliferative and anti-apoptotic programs during disease transformation.

To assess if different pro- and anti-apoptotic relatives are expressed in distinct tumor subpopulations, we performed unsupervised clustering of leukemic cells and searched for BCL-2 family genes among the biomarkers of each cluster. Five different clusters were identified. Cluster 4 was enriched in cells expressing *HRK*. Cluster 1 had a higher *BCL2A1* expression as compared to the others, whereas cluster 3 displayed higher *BID* expression (Fig. 5D, Supplementary Fig. 4B). Interestingly, the *HRK*^high^ cluster underwent contraction upon disease transformation, confirming the downmodulation of this sensitizer in the RS-like phase (Fig. 5D).

To uncover differences in gene regulatory network (GRN) between CLL and RS-like phase, we applied SCENIC workflow [26] to our dataset and identified 10 differentially expressed regulons. Each regulon corresponds to an individual TF with its target gene set. Six regulons were upregulated in the CLL phase (TCF4, JUND, ENO1, BCLAF1, SPIB, and SMARCB1), and 4 in the RS-like phase (ZNF274, JUN, FLI1, and BACH2) (Fig. 5E, Supplementary Fig. 4C). These GRN-based differences enabled a clear segregation of CLL and RS-like timepoints (Supplementary Fig. 4A). Importantly, the CLL-specific SPIB1, BCLAF1, and SMARCB1 were identified as putative TFs activating *HRK* and *PMAIP1* expression in the CLL phase (Fig. 5F).

To evaluate if the loss of pro-apoptotic sensitizers could be a recurrent theme during high-grade evolution, we manually interrogated two publicly available RNA sequencing datasets from matched CLL-RS samples [3, 4]. The dataset from Nadeu and colleagues included single-cell results from 4 CLL patients transformed into DLBCL-type RS. Three and 2 out of 4 patients downregulated *PMAIP1* and *HRK* during disease transformation, respectively. Paradoxically, the pro-apoptotic activators *BCL2L11* (encoding BIM) and *BID* were more likely to be upregulated rather than suppressed during RS, and the effector *BAX* showed a heterogeneous pattern of modulation across patients (Supplementary Fig. 4D). Dataset from Parry and colleagues, which included bulk RNA sequencing results from 5 cases, showed an overall downregulation of pro-apoptotic sensitizers in RS, with *BMF* being the only one reaching statistical significance (Supplementary Fig. 4E). Additionally, the pro-apoptotic sensitizer NOXA was nearly absent at the protein level in our series of RS-PDXs (Supplementary Fig. 4F).

Because transcriptional deregulation of PUMA drives acquired resistance to venetoclax in CLL [27], we assessed its expression in indolent and aggressive disease phases. Our transcriptomic analysis together with Nadeu’s and Parry’s data showed no difference in terms of *BBC3* (PUMA) expression between CLL and RS (Fig. 5B, Supplementary Fig. 4D, E). qPCR and western blot revealed similar PUMA expression in our RS models and CLL samples as well (Supplementary Fig. 5A, B). Immunohistochemical assessment of 8 CLL and 4 RS biopsies, including 3 matched CLL-RS samples, showed an unexpectedly higher expression of PUMA in RS compared to CLL (Supplementary Fig. 5C), suggesting that CLL cases acquiring resistance to BCL-2 antagonism [27] and those transforming into RS might modulate PUMA in an opposite manner.

Altogether, high-grade transformation is accompanied by transcriptional downregulation of selected pro-apoptotic sensitizers, while leaving unchanged or even paradoxically upregulated the pro-apoptotic effectors and activators.

### Pro-apoptotic effectors and activators are less likely to colocalize with mitochondria in RS

**Fig. 6:**
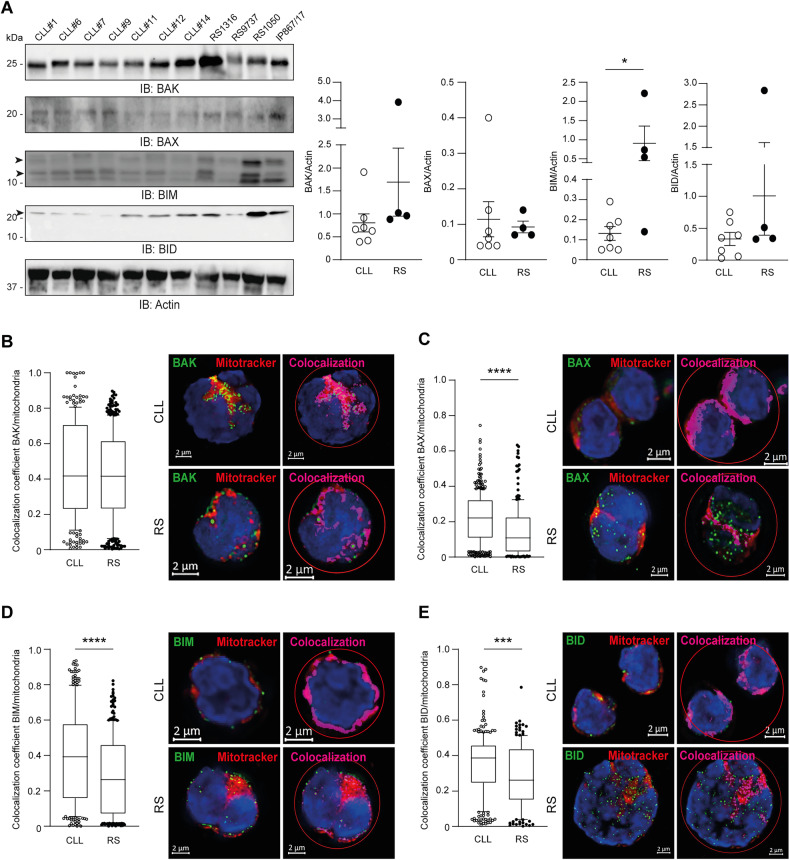
Subcellular localization of selected pro-apoptotic effectors and activators in CLL and RS. **A** Western blot analysis for the indicated pro-apoptotic effectors and activators in CLL and RS. Dot plots represent intensity of proteins bands in CLL (open circles) and RS (solid circles). Band intensities were measured using Image Lab and normalized on Actin. Data are reported as mean ± SEM. Unpaired Student *t*-test. *P < .05. (B-E) Four primary CLL samples and 3 RS-PDX models (RS9737, RS1316, and RS1050) were subjected to fluorescence microscopy to analyze the subcellular localization of pro-apoptotic effectors and activators. Each sample was stained with MitoTracker Deep Red FM to identify mitochondria, and with fluorescent monoclonal antibodies specific for the indicated pro-apoptotic proteins: **B** BAK, **C** BAX, **D** BIM, **E** BID. Samples were counterstained with DAPI. After selecting the field of interest (red circle), colocalization analysis was performed using Costes thresholding strategy automatically provided by the Zen module. Colocalization coefficients (=number of pixels colocalized with MitoTracker/total number of pixels occupied by the pro-apoptotic protein) of each Z-stack section of 8 CLL cells (2 for each patient) and 8 RS cells (3 for RS9737 and RS1316, and 2 for RS1050) were plotted (BAK: CLL *n* = 258 and RS *n* = 351; BAX: CLL *n* = 399 and RS *n* = 215; BIM: CLL *n* = 257 and RS *n* = 270; BID: CLL *n* = 273 and RS *n* = 155). Whiskers represent 10–90 percentile. Unpaired Student *t*-test, ****P < .0001; ***P < .001. Scale bar: 2 μm.

Western blot analysis showed heterogeneous expression of BAK, BAX, and BID in RS samples, with no significant difference when compared to CLL. Moreover, the activator BIM was even more expressed in RS with respect to CLL (Fig. 6A). During homeostasis, part of these molecules localizes at the OMM and part is constrained within the cytosol. The ratio between their cytosolic and mitochondrial fraction inversely correlates with chemosensitivity in human cancers [28, 29]. To evaluate whether subcellular localization of effectors and activators differ between CLL and RS, we stained these pro-apoptotic members with specific monoclonal antibodies and used fluorescence microscopy to quantify the proportion that colocalized with mitochondria. Colocalization coefficient was automatically calculated for each Z-stack section of randomly selected CLL and RS cells. In line with previous data, BAK had a higher degree of colocalization with mitochondria than BAX in both disease conditions [30]. Importantly, while BAK displayed similar colocalization coefficients in CLL and RS cells (*P* = .11), BAX, BIM and BID were less likely to colocalize with mitochondria in RS as compared to CLL (*P* < 0.0001; Fig. 6B–E), suggesting high-grade cells need a second hit to bring these pro-apoptotic molecules onto the mitochondria for apoptosis induction.

### RS mitochondria exhibit reduced cristae width

**Fig. 7:**
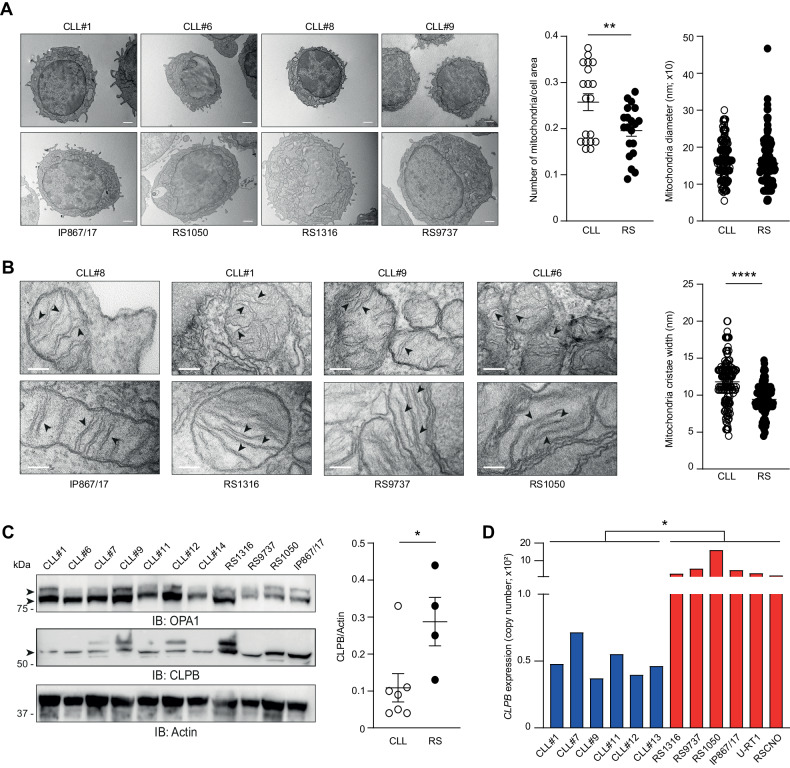
Ultrastructure of CLL and RS mitochondria. **A** Representative electron micrographs of CLL and RS cells for each analyzed sample. Original magnification: ×8900. Scale bar: 500 nm. Mitochondria were manually counted in 19 CLL (5 cells for #1, 5 for #6, 5 for #8, and 4 for #9) and 20 RS cells (5 cells for each model), and results were represented as number of mitochondria divided by cell area. Unpaired Student t test; means ± SEM. **P < .01. Mitochondrial diameter (minor axis length) was also measured in 191 and 200 mitochondria from the CLL and RS groups, respectively. **B** Representative electron micrographs of mitochondria from the indicated CLL and RS samples. Black arrowheads indicate examples of mitochondrial cristae in CLL and RS cells. Maximal cristae width was quantified on micrographs of 50 randomly selected mitochondria from 14 CLL and 14 RS cells (*n* = 141 cristae each group). Original magnification: x56000. Scale bar: 100 nm. Unpaired Student *t*-test; means ± SEM. ****P < 0.0001. **C** Western blot analysis for OPA1 and CLPB in CLL and RS samples. Dot plots represent intensity of proteins bands in CLL (open circles) and RS (solid circles). Band intensities were measured using Image Lab and normalized on Actin. Data are reported as mean ± SEM. *P < 0.05. **D** qPCR analysis of *CLPB* in the indicated RS and CLL samples. Unpaired Student *t*-test. *P < .05.

Cyt c is retained within the mitochondrial intermembrane space until pro-apoptotic stimuli reach the threshold for OMM permeabilization. The width of mitochondrial cristae has been demonstrated as an additional regulator of intrinsic apoptosis [31–33]. While wide cristae allow rapid cyt c leakage once OMM is permeabilized, tight cristae entrap cyt c within the intermembrane space, thus hindering downstream pro-apoptotic events [33]. Given the different propensity to release cyt c, we hypothesized that CLL and RS mitochondria could harbor different cristae conformations. To test this, we performed TEM on 4 CLL samples and 4 RS-PDX models and focused the analysis on mitochondrial morphometry. Compared to CLL, RS cells had a lower number of mitochondria per surface unit, with no differences in terms of diameter (Fig. 7A). Importantly, the width of mitochondrial cristae was reduced in RS cells with respect to CLL cells (mean cristae width: 11.7 ± 0.26 nm in CLL versus 9.41 ± 0.17 nm in RS, *P* < 0.0001; Fig. 7B). Expression of OPA1 and CLPB, two mitochondrial proteins involved in cristae tightening [31–33], was evaluated by western blot. While OPA1 levels did not change between CLL and RS, CLPB was more expressed in RS (Fig. 7C). This was consistent with qPCR results showing higher *CLPB* transcript in RS (Fig. 7D). These findings suggest the acquisition by RS cells of a mitochondrial morphology that further favors apoptosis evasion.

## Discussion

Through a comparative analysis of apoptosis regulation in CLL and RS, we provide evidence that the anti-apoptotic strategies adopted by leukemic cells evolve during disease transformation. RS mitochondria acquired the ability to retain cyt c even when exposed to high amounts of pro-apoptotic peptides, thereby having a low apoptotic priming, and are less dependent upon BCL-2 for survival as compared to CLL. In some RS cases (e.g., RSVR3, RS1050, RS1316), MCL-1, BCL-xL, and BFL-1 exerted a prominent role in protecting cancer cells from apoptosis, suggesting their direct targeting might be effective and outperform BCL-2 antagonism. Conversely, other RS samples (e.g., U-RT1, IP867/17) did not harbor any specific anti-apoptotic dependency and did not undergo apoptosis following incubation with MCL-1 or BCL-xL antagonists. For these samples, inhibition of upstream oncogenic pathways could be a valuable approach to increase the apoptotic priming and to timely expose anti-apoptotic dependencies for effective combination strategies. Single-cell transcriptomics of a matched CLL-RS case together with results from two independent studies [3, 4] indicated the class of pro-apoptotic sensitizers, particularly HRK and NOXA, was recurrently downregulated at transcriptional level during high-grade transformation. Of note, epigenetic suppression of HRK has been implicated in the pathogenesis of several human cancers including high-grade lymphomas [34, 35], and NOXA has been identified as a rheostat for venetoclax sensitivity in different types of blood malignancies [36–38]. Surprisingly, we found no evidence of downregulation for pro-apoptotic effectors and activators, which were in some cases even more expressed in RS compared to CLL. This may suggest for these molecules some tumor-beneficial, non-apoptotic roles ultimately fostering proliferative programs and metabolic rewiring [39, 40].

Despite having comparable expression in CLL and RS, BAX as well as BIM and BID displayed a lower level of colocalization with mitochondria in RS. Moreover, decreased apoptotic priming of RS cells parallels the tightening of their mitochondrial cristae. The shape of mitochondrial cristae has long been recognized as a key determinant of mitochondrial function, with narrow cristae favoring the assembly and stability of respiratory chain complexes [41]. Functional and transcriptomic data are piling up about the upregulation of oxidative phosphorylation in RS [3], a metabolic signature that may find in cristae tightening its morphological correlate. While in vitro manipulation of cristae width has been shown to tune the apoptotic priming of cancer cells [33], our work is the first one demonstrating that changes in cristae shape may naturally occur during high-grade transformation, a condition eventually furthering the metabolic efficiency and the anti-apoptotic attitude of RS mitochondria.

Our functional results are in line with the clinical results of BCL-2 antagonism. Objective responses have been observed in 75% of CLL patients treated with venetoclax as single agent, with a median progression-free survival of 30.2 months [42]. In contrast, only 3 out of 7 RS patients were sensitive to venetoclax in the phase I first-in-human study, and only in one patient the duration of response exceeded 24 months [43]. Though disappointing as monotherapy in RS, venetoclax deserves further exploration in combination with agents that increase the apoptotic priming and the BCL-2 dependence of RS cells, such as chemotherapy and BTK inhibitors [44, 45].

Moving beyond clinical observations, the reduced apoptotic priming of RS can be ultimately considered as a large step away from B-cell physiology. Several immunology studies indicate that fully functional intrinsic apoptosis is required to delete autoreactive clones during B-cell ontogenesis and eliminate activated B cells producing low-affinity antibodies [46–48]. Indeed, normal lymphocytes usually have a high apoptotic priming [49]. From this viewpoint, CLL cells appear strikingly like their normal counterpart, still preserving an effective commitment to apoptosis that is only controlled by the function of one, or at most two, anti-apoptotic proteins [12]. This relatively simple way to evade apoptosis is nowadays successfully beaten by venetoclax. In sharp contrast, RS cells implement a series of mechanisms, including downmodulation of sensitizers, cytosolic relocation of pro-apoptotic members, cristae tightening, and upmodulation of heterogeneous non-BCL-2 anti-apoptotic dependencies, that render apoptosis evasion more robust in nature and hence much harder to dismantle.

A potential limitation of our study is that all primary RS samples had leukemic involvement by large B cells and were collected from peripheral blood. Although this allowed a fairer comparison with CLL samples, collected from peripheral blood as well, we acknowledge that the majority of RS cases encountered in the clinic have no leukemic spread and manifest as fast-growing nodal or extranodal masses. Moreover, one of our primary samples was compatible with RS-like transformation, a transient state triggered by abrupt ibrutinib interruption and pathogenetically distinct from true RS. Therefore, additional studies involving RS cells collected from tumor masses are needed to validate and expand our findings.

In conclusion, our work reveals a functional shift away from apoptosis commitment during high-grade transformation, defining poor apoptotic priming and low BCL-2 dependency as two novel functional hallmarks of RS. Moreover, we have identified multiple mechanisms potentially involved in apoptotic priming collapse. A broader understanding of these processes might open the way to more rational strategies for directly targeting apoptosis in RS and possibly other types of aggressive lymphomas.

## Methods

### Patient samples and RS models

The study was undertaken in accordance with the principles of the Declaration of Helsinki and was approved by the local ethics committee (protocol #1747CESC). All enrolled patients provided written informed consent. Seventeen clinically and biologically annotated CLL samples and 4 RS samples in leukemic phase were obtained from the University Hospital of Verona and from the Hematology Unit, Hospital S. Croce e Carle, Cuneo. All CLL patients fulfilled the current criteria for CLL diagnosis [50]. RS diagnosis was always confirmed by bone marrow or lymph node histology. All primary RS and RS models were clonally related to prior CLL. In all primary RS cases enrolled in this study, large B cells were detected at peripheral blood smear and collected for downstream analyses. Large B cells represented 80%, 68%, 85% and 65% of the total leukemic cells in RSVR1, RSVR2, RSVR3, and RSCNO, respectively. RS-like syndrome induced by ibrutinib interruption (RSVR2) was defined by the appearance of large B cells in peripheral blood, accompanied by clinical (B symptoms) and laboratory signs (LDH > 2 times of upper normal limit) of disease transformation. Peripheral blood mononuclear cells (PBMC) were viably frozen in fetal bovine serum (FBS) supplemented with 10% DMSO until thawing. PBMC from CLL patients were composed for more than 90% of leukemic cells. Four previously characterized RS-PDX models, maintaining the same phenotypic and genetic features of the primary tumors, were utilized [51, 52]. Three of them (RS9737, RS1316, and RS1050) were established from patients who had received chemotherapy and/or targeted agents, whereas the fourth (IP867/17) was established from a treatment-naïve patient. The local animal care and use committee approved this study. Mice were treated according to the European guidelines for animal use in scientific research, and with the approval of the Italian Ministry of Health (authorization #664/2020; protocol #CC652.136). For in vitro experiments, RS cells were withdrawn from tumor masses of PDX models and cryopreserved until use. U-RT1 [53] and DG-75 [54] (DSMZ, Braunschweig, DE) cell lines were cultured in RPMI supplemented with 10% FBS. Cell lines were authenticated routinely and Mycoplasma free.

### BH3 profiling and dynamic BH3 profiling

BH3 profiling was chosen over other methods that investigate anti-apoptotic dependencies due to its short incubation time. CLL and RS samples have different rates of spontaneous apoptosis ex vivo. Therefore, cytotoxicity results derived from prolonged incubation with small molecules are biased by the different proclivity to commit spontaneous apoptosis across samples in culture. BH3 profiling was performed as previously described by Ryan and colleagues [18], with minor modifications. Thawed cells were resuspended at a concentration of 2 × 10^6^/mL in PBS/Zombie Yellow viability dye (1:500) (BioLegend, San Diego, CA) for 30 min at room temperature (RT). After washing with PBS/2% FBS, cells were resuspended in the same buffer and stained for 15 min RT with anti-human CD5-FITC and anti-human CD19-V450 (Becton Dickinson, San Jose, CA) monoclonal antibodies. After washing in PBS, cells were resuspended in MEB2 buffer (150 mM mannitol, 10 mM HEPES, 150 mM KCl, 1 mM EGTA, 1 mM EDTA, 0.1% IgG-free BSA, pH 7,5 with KOH) at 4 × 10^6^/mL. To prepare BH3 profiling reactions, 100 μL of MEB2/20 μg/mL digitonin was added to flow cytometry tubes. Synthetic peptides (Peptide Synthetics, Fareham, UK) or small molecules (venetoclax or A-1331852, from Selleckchem, Munich, DE) were added at different concentrations to each tube and 100 μL of cell suspension was dispensed in each tube, in duplicate. Tubes were incubated at 25 °C in the dark in gentle agitation for 60 min. Cells were fixed for 10 min by the addition of 67 μL PBS/4% formaldehyde methanol-free (Thermo Fisher Scientific, Waltham, MA), quenched for 15 min by 67 μL N2 buffer (1.7 M Tris base, 1.25 M glycine, pH 9.1) and stained overnight at 4 °C with 40 μL of 10X intracellular staining buffer (10% BSA, 2% Tween20 in PBS) containing anti-human cytochrome c-Alexa Fluor 647 monoclonal antibody (1:400, BioLegend). A total of 30,000 CD5^+^/CD19^+^ cells were acquired on a FACSCantoII cytometer (Becton Dickinson) and analyzed by FlowJo 9.9.6 software (Tree Star, Ashland, OR). Hierarchical gating was used to accurately identify cells of interest and to exclude doublets and dead cells (Supplementary Fig. 1). Following morphological gate (FSC-A/SSC-A) and doublets exclusion (FSC-A/FSC-H), the remaining dead cells were excluded by gating on Zombie Yellow negative cells. In primary samples, the population of interest was identified by the immunological gate on CD5^+^/CD19^+^ cells. In RS-PDX models, the population of interest was identified by the immunological gate on CD19^+^ cells. Because RS cells did not harbor specific surface markers and morphological details were lost upon digitonin exposure, large RS cells could not be separated from residual CLL cells during the BH3 profiling analysis of primary samples. An inert peptide (PUMA2A) was used to define full cyt c retention (negative control) while 25 μM alamethicin (Abcam, Cambridge, UK), served as a complete cyt c release control (positive control). The effect of peptides and drugs was calculated by using median fluorescence intensity of the cyt c staining, as follows:

$$\% \,{\rm{cyt}}\; {\rm{c}}\; {\rm{loss}}={{\rm{MFI}}}_{{\rm{sample}}}{{\mbox{-}}}{{\rm{MFI}}}_{{\rm{alamethicin}}}/{{\rm{MFI}}}_{{\rm{PUMA}}2{\rm{A}}}{{\mbox{-}}}{{\rm{MFI}}}_{{\rm{alamethicin}}}$$

Dose–response curves for BIM peptide were generated with GraphPad Prism 9 using log(inhibitor) vs. response–variable slope (four parameters). The concentrations of BH3 peptides used for the generation of the heatmap in Fig. 1B are detailed in Supplementary Table 2.

For dynamic BH3 profiling (DBP) [55], cells were plated in RPMI-1640/penicillin-streptomycin/10% FCS and treated with a panel of 9 pathways inhibitors, each at the concentration of 1 μM for 16 h. Cells were collected, washed in PBS, and subjected to BH3 profiling using BIM (0.005 μM for RS1050 and RS9737, 0.05 μM for RS1316 and IP867, and 1 μM for U-RT1) or PUMA2A peptides. The concentration of BIM was chosen to obtain a basal (non-drug induced) cyt c release ranging from 10 to 30%. To calculate the percent change in mitochondrial priming (delta priming), we first determined the percentage of cyt c loss for cells exposed to pro-apoptotic peptides alone as for standard BH3 profiling. Finally, the following formula was applied:

$${\rm{delta\; priming}}= \% \,{{\rm{cyt\; c\; loss}}}_{{\rm{drug}}}- \% \,{{\rm{cyt\; c\; loss}}}_{{\rm{DMSO}}}$$

For the extended DBP shown in Fig. 4G, the indicated RS-PDX models were treated with 1 μM copanlisib for 3 or 6 h and then subjected to BH3 profiling using BAD, MS-1, HRK, or FS-1 peptides, each at the concentration of 1 μM.

### Real-time monitoring of caspase 3/7 activation

Induction of apoptosis upon venetoclax treatment was monitored over time using the CellEvent^TM^ Caspase-3/7 Green Assay Kit (Thermo Fischer Scientific). Regarding the experiment in Fig. 2B, PBMC from CLL #6, #7, #9, #13 and RS cells from each of the RS-PDX models were dispensed in polylysine-coated 24-well plates at a density of 10^6^ cells/well, and treated with DMSO or 50 nM venetoclax. For the experiments shown in Fig. 4A, RS models were treated with DMSO, 50 nM A-1331852 or 5 μM S63845. Kinetics of Caspase-3/7 cleavage was evaluated by time-lapse video microcopy with a Zeiss AxioObserver 7 inverted wide-field microscope, equipped with thermostatic chamber, Colibri 7 fluorescent LED illumination, full motorized stage, Hamamatsu ORCA-Flash4.0 V3 Digital CMOS camera, set at 8 output bit depth, and the Zeiss ZEN 2.6 time-lapse module (Oberkochen, DE). Plates were kept at 37 °C in 5% CO_2_ humidified atmosphere; movie acquisition was with a 40x Plan Apochromatic objective (AN 0,65) corresponding to an acquisition area of 1.2 × 10^5^ mm^2^, with a cell density of 500 cell/acquisition area. Each field was acquired in brightfield and fluorescent light illumination (503/530 nm ex/em). Exposure time for fluorescent light was set at 500 msec and left unchanged for the entire duration of the experiment. Time-lapse imaging was for 7 h, with a 30 min time frame. Frame-by-frame image analysis was performed, without image preprocessing, with the Zeiss ZEN 2.6 image analysis module, allowing automatic cell segmentation, recognition and pixel intensity quantification. Data were expressed and plotted as normalized number of Caspase-3/7 positive cells over time.

### Single-cell RNA sequencing of patient undergoing RS-like transformation

PBMC from an individual patient in two different disease phases (CLL #14 and the RS-like phase RSVR2) were isolated and viably frozen. After thawing, cells were resuspended in RPMI supplemented with 5% FBS to achieve a final concentration of 1000 cells/mL. Ten thousand live cells were loaded onto the Chromium controller to recover 4000 single-cell GEMs per inlet uniquely barcoded. After cDNA synthesis, sequencing libraries were generated. Final 10× library quality was evaluated using the Fragment Analyzer High Sensitivity NGS kit (Agilent Technologies, Santa Clara, CA) and then sequenced on the Illumina NextSeq500 (Illumina, San Diego CA) generating 75 base pair paired-end reads (28 bp read1 and 91 bp read2) at a depth of 50,000 reads/cell. Raw base call (BCL) files were processed using Cell Ranger (10× Genomics) from PartekFlow software to obtain a unique molecular identifier (UMI) count table. To perform these steps, *Homo Sapiens (Human)* reference data (hg38 Feb 3, 2022) was downloaded from the 10× official website. Next, the dataset was analyzed using the PartekFlow software. As first, we filtered out low-quality cells, such as doublets, damaged cells, or those with too few reads, evaluating the number of read counts per cell (600–15000), the number of detected genes per cell (200–4000), the percentage of mitochondrial reads per cell (0–10), and the percentage of ribosomal counts per cell (10–60) After quality control, we obtained a total of 7762 cells. Following the recommended normalization step, through which counts were normalized and presented in logarithmic scale in CPM (count per million) approach, features were filtered excluding genes that are expressed by any cells in the dataset, thus obtaining a total number of 15296 genes. Batch correction was provided using the general linear model task, available in the PartekFlow. Garnett Classification was used to identify B cells (*n* = 6271), on which UMAP dimensional reduction and unsupervised clustering were performed. Clusters biomarkers were automatically computed by the software performing a Student’s *t*-test on the selected attribute, comparing one subgroup at a time with all the others combined. Gene-specific analysis (GSA) and GSEA were performed on the cell population of interest using PartekFlow plugins. Similarly, we used PartekFlow software to run, after the scaling expression, the trajectory analysis, based on Monocle3 R package. We thus identified states and branch points, also calculating pseudotime values. Study of gene regulatory network was performed by applying SCENIC R-based package to our dataset [26].

### Western Blots

Primary CLL (10–20 × 10^6^), RS-PDX (2 × 10^6^), and U-RT1 (2 × 10^6^) cells were lysed and protein concentration measured using the Bradford Protein Assay (Bio-Rad, Milan, Italy), according to the manufacturer’s protocol. Proteins were resolved using a 12% MiniPROTEAN® TGX™ Precast Protein Gels (Bio-Rad) and transferred into 0.2 μm nitrocellulose Trans-Blot Turbo Transfer membrane using the Trans-Blot Turbo Transfer System (all from Bio-Rad). Membranes were blocked with 5% non-fat dry milk in a Tris buffer 0.1% Tween prior to primary antibody incubation (overnight; 4 °C) followed by a secondary antibody HRP-conjugated. All the antibodies used are listed in Supplementary Table 3. Blots were incubated with the Clarity or Clarity Max Western ECL substrates and images acquired with a ChemiDoc XPS+ imaging system (Bio-Rad). Densitometric quantification was performed using the Image Lab Bio-Rad software and bands were normalized on the β-actin, used as a loading control. Original blots are provided in Supplementary Fig. 6.

### Real-time quantitative polymerase chain reaction (qPCR)

RNA was extracted from primary CLL (1–10 × 10^6^), RS-PDX (5 × 10^5^), and U-RT1 (5 × 10^5^) cells using the RNeasy Plus Mini Kit (Qiagen, Milan, Italy), and retro-transcribed to cDNA using the High-Capacity cDNA Reverse Transcription Kit (Applied Biosystems Thermo Fisher, Milan, Italy), according to the manufacturer’s instructions. qPCR was performed using 4 ng cDNA with iTaq Universal Probes SuperMix (#1725134, BioRad) and gene-specific probes for *B2M* (Hs00984230_m1), *BCL-2* (Hs00608023_m1), *BCL*-*xL* (Hs00236329_m1), *MCL*-1 (Hs01050896_m1), *BBC3* (Hs00248075_m1), *CLPB* (Hs00229376_m1), *BCL2A1* (Hs00187845_m1), all from Thermo Fisher Scientific using the CFX384 Real-Time System (Bio-Rad). Reactions were performed in triplicate. For each gene, expression levels were computed as a ratio of the number of copies of the target gene over 10^5^ copies of *B2M*.

### Flow cytometry assessment of BTK activation and MCL-1 expression

BTK activation was measured by means of tyrosine 223 phosphorylation. Briefly, 0.5 × 10^6^ cells were fixed in 100 μL 4% formaldehyde for 30 min at 4 °C. Cells were washed in PBS and suspended in 50 μL of permeabilization buffer (PBS + 5% FBS + 0.5% saponin) containing PE-conjugated anti-pY223-BTK antibody (BD Biosciences) or isotype control (Merck, Rahway, NJ) for 30 min at 4 °C. Cells were washed and suspended in 200 μL ice-cold PBS and analyzed by flow cytometry. For MCL-1 expression, 1 × 10^6^ cells were stained with Fixable Viability Stain 780 (BD, 5665388) in PBS for 15 min at room temperature. After washing cells in Facs Buffer (PBS1X + 2%FBS) and staining with CD19-PE-Cy7 (SJ25C1 clone, 557835, BD) for 15 min at RT, cells were washed with permeabilization buffer (00-5523, eBioscience) and fixed in 100 μL 0.4% formaldehyde for 30 min at 4 °C. Intracellular staining was performed by using anti-Mcl-1 Alexa-Fluor 488-conjugated antibody (D2W9E clone, BK583265 CST, Euroclone) in permeabilization buffer for 30 min at 4 °C. Cells were washed in permeabilization buffer and analyzed by flow cytometry.

### PUMA immunohistochemistry

Tissue sections from formalin-fixed paraffin-embedded (FFPE) primary RS biopsies and CLL-RS matched sample biopsies were stained with an anti-PUMA rabbit polyclonal antibody (ab9645, Abcam, Cambridge, UK), followed by an anti-rabbit HRP-conjugated antibody and 3,3’-diaminobenzidine (EnVision™ System, Dako, Glostrup, DK) to visualize the reaction. Anti-PUMA specificity on tissues was tested on reactive lymph nodes and breast cancer samples. Immunohistochemical expression of PUMA was evaluated semiquantitatively as percentage of positive cells (0%; 0–25%; 25–50%; 50–75%: >75%) and as staining intensity (1+, 2+, 3+).

### Fluorescence microscopy and colocalization analysis

CLL and RS-PDX cells were stained with 300 nM MitoTracker Deep Red FM (Thermo Fisher Scientific) for 30 min at 37 °C. Cells were fixed with formaldehyde 0.4% for 20 min and washed with PBS/5% FCS. Cells were permeabilized with PBS/0.1%Triton X-100 for 7 min at 4 °C. After washing and blocking for 15 min at 4 °C with PBS/5% FCS, cells were incubated for 1 h at 4 °C with mouse anti-human BAX and BID (BD Biosciences, Franklin Lakes, NJ and Proteintech, Rosemont, IL, respectively) and rabbit anti-human BAK and BIM (Abcam) monoclonal antibodies. After washing, cells were incubated for 40 min at 4 °C with goat anti-mouse or goat anti-rabbit IgG AlexaFluor488 antibodies (Abcam). After washing, cells were stained with 1 μg/mL DAPI, transferred onto a glass slide, and mounted with Fluoro Gel with DABCO (Electro Microscopy Sciences, Hatfield, PA). Images were acquired with a wide field Zeiss AxioImager Z.2 deconvolution microscopy setting, equipped with Colibri 7 fluorescent LED illumination, motorized 3D scanning stage, and Hamamatsu ORCA-Flash4.0 V3 Digital CMOS camera, set at 8 bit output depth. 512 × 512 pixel ROIs were acquired with a 100x Plan Apochromatic oil immersion objective (AN 1.46). Each field was acquired with triple fluorescent light illumination (385/30 nm ex. for DAPI, 475/36 nm ex. for Alexa Fluor 488 and 631/33 nm ex. for Cy5-Deep Red FM). Automatic 3D image scanning was according to the Nyquist-Shannon sampling theorem, by using the inline ZEN 3.5 Nyquist Calculator. 3D scans were, then, processed with Zeiss ZEN 3.5 by applying the advanced Zeiss Deconvolution (DCV) module. Image deconvolution was achieved by applying the Constrain Iterative algorithm, without auto-normalization to fully control the photon budget thus allowing full reassignment of photons from out-of-focus optical planes. Spectral linear unmixing was, finally, applied to remove overlapped spectral components and background noise. Deconvolved and unmixed 3D stacks were rendered and analyzed with the ZEN 3.5 Colocalization module. Colocalization of pro-apoptotic proteins with mitochondria was quantified in each slice using Costes thresholding strategy [56], automatically provided by the Zen module. Colocalization coefficients (calculated as ratio of number of pixels colocalized with Mitotracker over total number of pixels occupied by the pro-apoptotic protein) of each section of 8 CLL cells from 4 individual patients and 8 RS cells from 3 RS-PDX models (RS9737, RS1316, RS1050) were plotted.

### Transmission electron microscopy

For ultrastructural examination, cell pellets of 4 CLL patients (#1, #6, #8, and #9) and 4 RS-PDX models were fixed for 1 h in 2% glutaraldehyde in 0.1 M phosphate buffer and, after washing, postfixed for 1 h in 1% OsO_4_ diluted in 0.2 M K_3_Fe. Subsequently, samples were dehydrated in graded concentrations of acetone and embedded in a mixture of Epon and Araldite (Electron Microscopic Sciences, Fort Washington, PA). Ultrathin sections were cut at 70 nm thickness on an Ultracut-E ultramicrotome (Reichert-Jung), stained with lead citrate, and observed on a Philips Morgagni 268 D transmission electron microscope operating at 80 kV (Fei Company, Eindhoven, NL), equipped with Megaview II camera for acquisition of digital images. The mitochondrial count was performed on micrographs at ×5600 of 19 randomly selected CLL cells (5 cells from #1, 5 from #6, 5 from #8, and 4 from #9) and 20 randomly selected RS cells (5 for each sample). The minor axis length was measured on micrographs at ×8900 of 191 CLL and 200 RS mitochondria from 20 different cells. The cristae width was measured on micrographs at x56000 of 50 randomly selected mitochondria from 14 cells in the CLL group and 50 mitochondria from 14 cells in the RS group, for a total of 141 cristae each condition. Measurements were taken using Emsis Radius 2 software and are expressed as mean ± SEM.

### Statistics

Statistical analyses were conducted using GraphPad Prism 9. Unpaired two-tailed Student t test was used to compare unpaired groups. Unless otherwise specified, data are presented as means ± SEM. Differences were considered significant for *P* values <0.05.

### Supplementary information

Supplementary information file

## Data Availability

The datasets generated during the current study are available from the corresponding author on reasonable request.
