# Supplementary material for: Decreased apoptotic priming and loss of BCL-2 dependence are functional hallmarks of Richter’s syndrome
Source: Cell Death Dis. 2024 May 9;15(5):323. doi: 10.1038/s41419-024-06707-5 (PMC11082225; doi:10.1038/s41419-024-06707-5)
Supplement: Supplementary file 1 — Supplementary information file [file 41419_2024_6707_MOESM1_ESM.pdf]

Supplemental Table 1. Clinical and biological features of the CLL patients enrolled in this study and subjected to BH3 profiling

CLL #	Age (years)	Gender	Disease status	Previous therapies	IGHV	FISH	TP53	Genomic complexity
1	53	Male	TN	NA	M	Del13q	WT	No
2	53	Male	TN	NA	U	Normal	WT	No
3	60	Male	TN	NA	U	Normal	WT	No
4	59	Male	TN	NA	M	Del13q	WT	No
5	73	Male	TN	NA	NE	Del13q	WT	No
6	45	Male	TN	NA	U	Del13q, del 11q	WT	No
7	75	Male	TN	NA	U	Tris12	mutant	No
8	82	Female	TN	NA	NE	Tris12	WT	No
9	66	Female	TN	NA	U	Del13q	WT	No
10	74	Female	R/R	FCR, BR	U	Del11q	mutant	NE
11	61	Female	R/R	Ibr	U	Del11q, tris12, del17p	mutant	Yes
12	68	Female	R/R	BR	U	Del11q, tris12, del17p	mutant	Yes
13	59	Male	R/R	FCR	NE	Del13q	WT	No
14	53	Male	R/R	FCR, Ibr	U	Normal	WT	Yes
15	75	Male	R/R	FCR	M	Del13q	WT	No
16	70	Male	R/R	BR, Ibr	U	Del11q	WT	No
17	74	Female	R/R	BR, Ibr	U	Tris12	mutant	No

Abbreviations: TN: treatment-naïve; R/R: relapsed/refractory; NA: not applicable; NE: not evaluated; WT: wild type; BR: bendamustine-rituximab; Ibr: ibrutinib; FCR: fludarabine, cyclophosphamide, rituximab; M: mutated; U: unmutated; FISH: fluorescence in situ hybridization. FISH analysis was limited to 13q deletion, trisomy 12, 11q deletion, and 17p deletion. Genomic complexity is defined as 3 or more cytogenetic aberrations based on conventional metaphase karyotype analysis.

Supplemental Table 2. Concentrations of BH3 peptides used for BH3 profiling

BH3 peptides	Concentrations (μM)
BIM	10, 1, 0.1, 0.05, 0.01
BID	10, 1, 0.1, 0.01
BMF	100, 10, 1
PUMA	100, 10, 1, 0.1
BAD	100, 10, 1, 0.1
MS-1	10, 1
HRK	10, 1
FS-1	10, 1, 0.1

Supplemental Table 3: Antibodies used in Western Blot

Protein	Vendor	Catalog #	Species	Application
BAK	Cell Signaling Technology	6447S	Rabbit	Primary
BAX	Cell Signaling Technology	2772S	Rabbit	Primary
BCL-2	Cell Signaling Technology	4223S	Rabbit	Primary
BCL-xL	Cell Signaling Technology	2764S	Rabbit	Primary
BFL-1	Cell Signaling Technology	14093S	Rabbit	Primary
BID	Cell Signaling Technology	2002S	Rabbit	Primary
BIM	Cell Signaling Technology	2933	Rabbit	Primary
CLPB	Proteintech	15743-1-AP	Rabbit	Primary
MCL-1	Cell Signaling Technology	94296S	Rabbit	Primary
OPA1	Cell Signaling Technology	67589S	Rabbit	Primary
PUMA	Abcam	AB9645	Rabbit	Primary
NOXA	Calbiochem	OP180	Mouse	Primary
β-actin-HRP	Santa Cruz Technology	Sc-47778	Mouse	Primary
Anti-rabbit HRP	Cytiva	NA934V	Sheep	Secondary

Supplemental Figures

Supplemental Figure 1. BH3 peptides and gating strategy. (A) Binding affinity of BH3 peptides for each anti-apoptotic protein. (B) Concentrations of BH3 peptides used for the generation of the heatmap in Figure 1B. (C) Gating strategy for BH3 profiling. Debris and doublets were excluded. Live cells, defined as Zombie-negative cells, were gated. Final analysis was conducted on CD5+/CD19+ cells. The inert peptide PUMA2A was used as control for complete cyt c retention, whereas alamethicin was used as control for complete cyt c release.

Supplemental Figure 2. Clinical history of primary RS samples subjected to BH3 profiling. (A) Timeline showing the main biological features and the treatments received by RSCNO, RSVR1, RSVR2 and RSVR3 patients. FCR: fludarabine, cyclophosphamide, rituximab; BR: bendamustine, rituximab; Ibr: ibrutinib; Ven: venetoclax; R-CHOP: rituximab, cyclophosphamide, doxorubicine, vincristine, prednisone; RT: radiation therapy; scRNAseq: single-cell RNA sequencing. (B) Bone marrow histology of RSVR3, compatible with DLBCL-type RS. Magnification: x200. Scale bar: 50 µm.

Supplemental Figure 3. BH3 profiling of CLL cases stratified for *TP53* mutation. Patients with *TP53*^{MUT} harbored one or more mutations of *TP53*. One of them also had 17p deletion detected at FISH analysis. No patient with *TP53*^{WT} had 17p deletion. Apoptotic priming was derived by using 10 µM BIM. BCL-2 dependence was derived by subtracting the effect of HRK (specific for BCL-xL) from that of BAD, or by applying venetoclax 1 µM or 0.1 µM.

Supplemental Figure 4. Transcriptomics of CLL and RS. A-C Single-cell RNA sequencing of CLL14 (= CLL phase) and RSVR2 (= RS-like phase). (A) UMAP plot of single-cell transcriptome profiles distinguishing B cells and normal immune populations in merged

CLL14/RSVR2 (*Upper left*). B cells were segregated into CLL (blue) and RS-like (red) phase based on B-cell identity genes (*Upper right*) or regulatory networks (*Lower right*). (B) UMAP visualization of B cells (CLL phase + RS-like phase) showing the expression of cluster-enriched marker genes (*HRK*, *BCL2A1*, *BID*). Transcript levels are color-coded: green refers to high expression, black low expression. (C) tSNE plots of gene regulatory networks differently expressed in CLL and RS-like phase (D) Log fold change (LogFC) of the expression of the indicated BCL-2 family members in single cell-RNA sequencing data of 4 paired CLL/RS cases as reported in the Supplementary Tables 23a-d from Nadeu et al (*Nat Medicine*, 28, 2022). Each column represents an individual case. Each box is the LogFC of the indicated gene in RS versus CLL phase. BCL-2 family genes are grouped based on their function (pro-apoptotic effectors: *BAX and BAK1*; pro-apoptotic activators: *BCL2L11, BID, BBC3*; pro-apoptotic sensitizers: *PMAIP1, HRK, BAD, BMF*; anti-apoptotic genes: *BCL2, MCL1, BCL2L1, BCL2A1*). LogFC is color-coded: red indicates increased expression in the RS versus CLL phase; blue indicates decreased expression in the RS versus CLL phase. Colored boxes are all statistically significant. All non-statistically significant values are represented in grey. (E) LogFC of the expression of the indicated BCL-2 family members in bulk RNA sequencing of 5 paired CLL/RS cases as reported in the Supplementary Table 9a from Parry et al (*Nat Medicine*, 29, 2023). $*P < .01$. (F) Western blot analysis for NOXA in the indicated RS-PDX samples and U-RT1 cell line. U2932 is a DLBCL cell line that was used as positive control of NOXA expression. Actin was used as loading control.

Supplemental Figure 5. PUMA expression in CLL and RS. (A) qPCR analysis of *BBC3* (encoding PUMA) in the indicated CLL and RS samples. $P = .08$ CLL vs RS (B) Western blot analysis for PUMA in CLL and RS samples. (C) Representative immunohistochemistry images of PUMA expression in biopsies of CLL and RS patients (magnification indicated inside each panel). The first three rows are node biopsies from three different patients affected by CLL, CLL with increased paraimmunoblasts, and RS, respectively. The fourth row shows different biopsies from an individual patient along his disease course. Disease

phase and tissue of origin are indicated above each panel. LN: lymph node. Left graph shows PUMA intensity (+, ++, +++) and the percentage of PUMA-positive cells (0-25%, 25-50%, 50-75%, >75%) in all CLL and RS histological samples. Right graph shows the evolution of PUMA intensity in 3 matched CLL-RS histological samples.

Supplemental Figure 6. Original western blots.

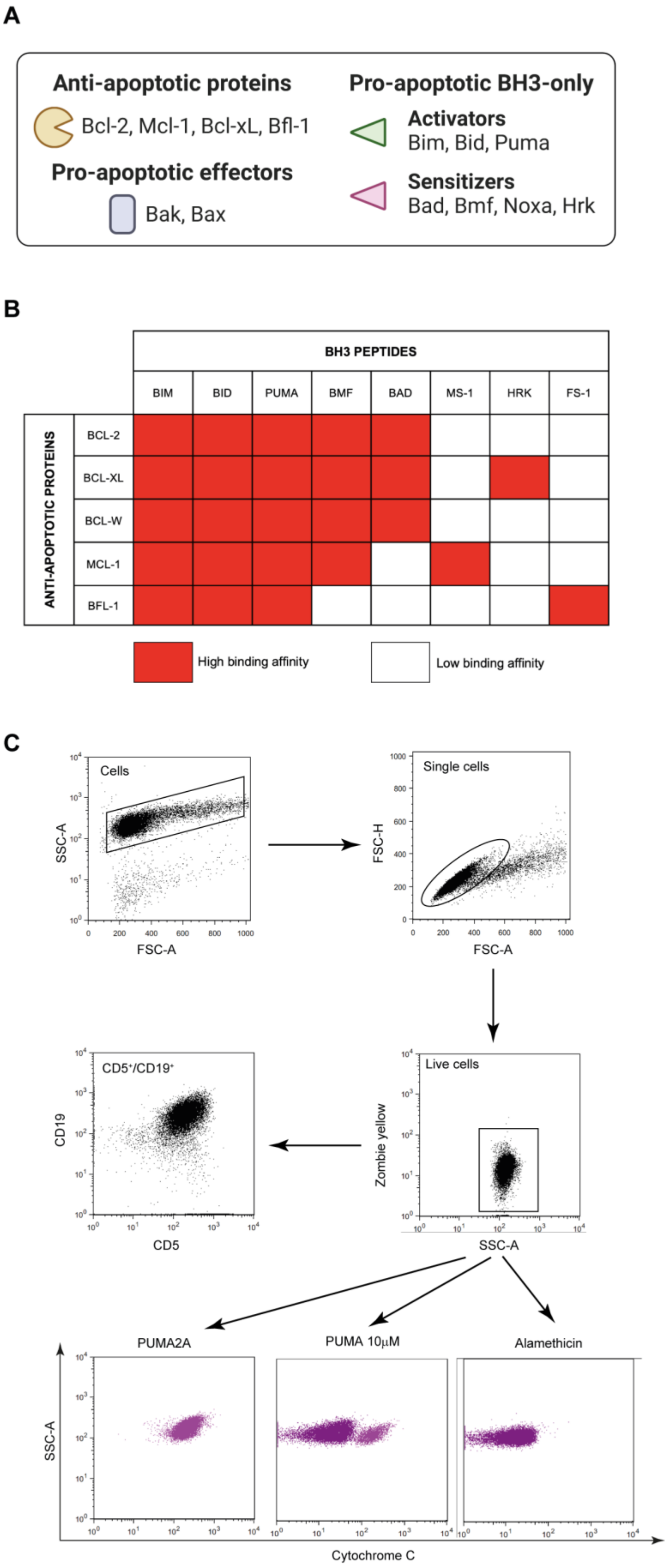

Supplemental figure 1

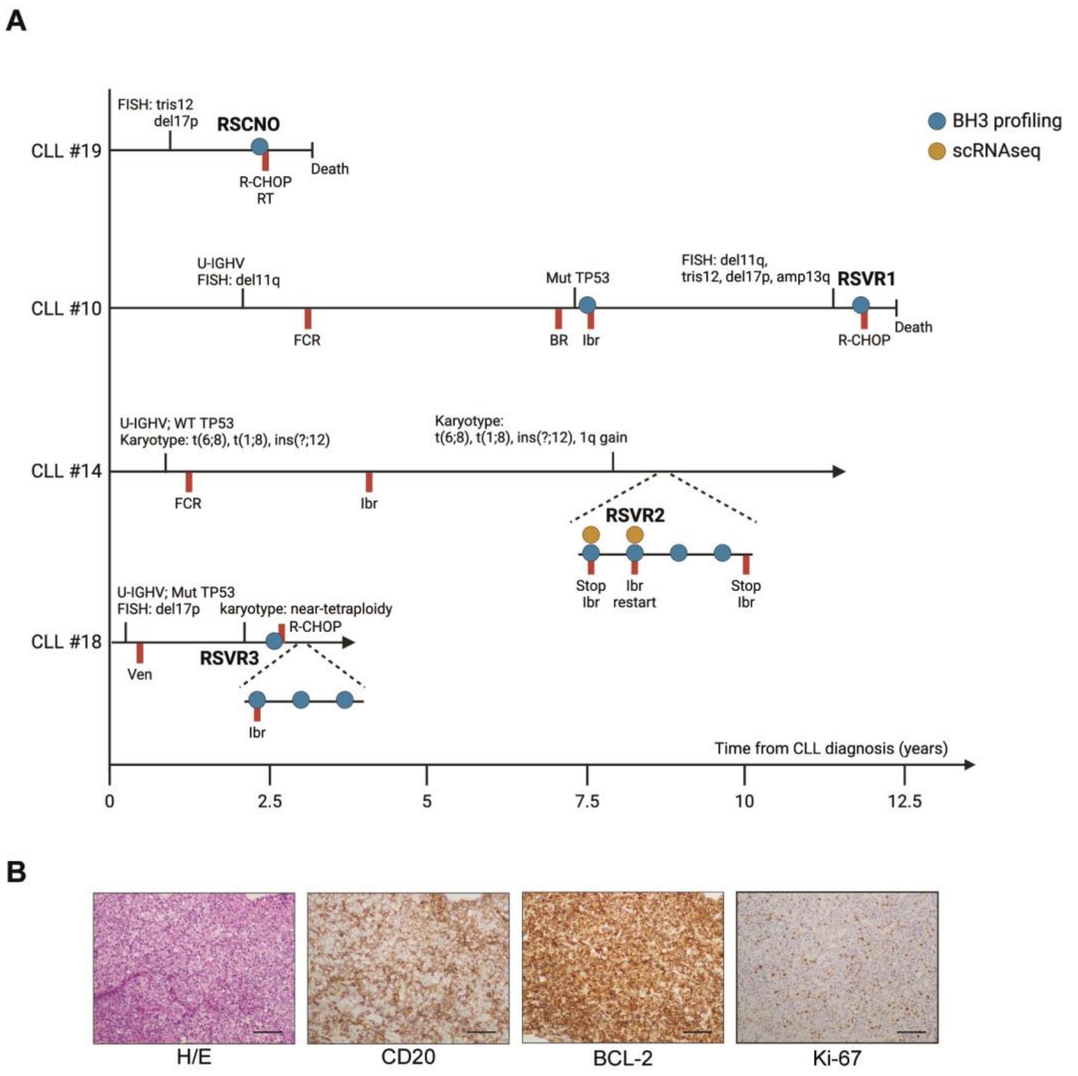

Supplemental Figure 2

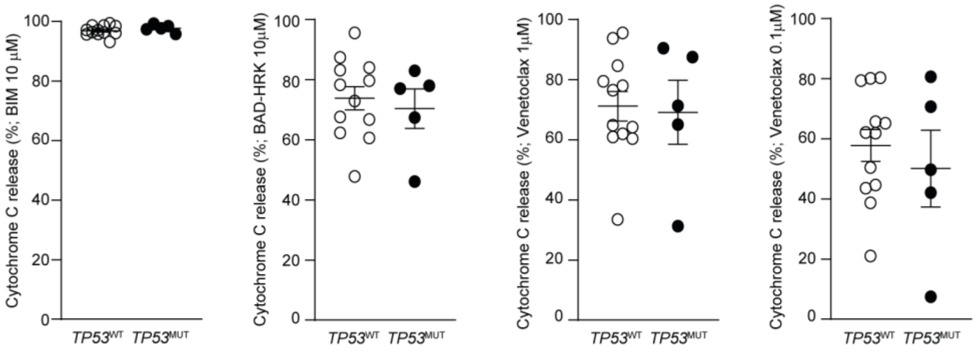

Supplemental figure 3

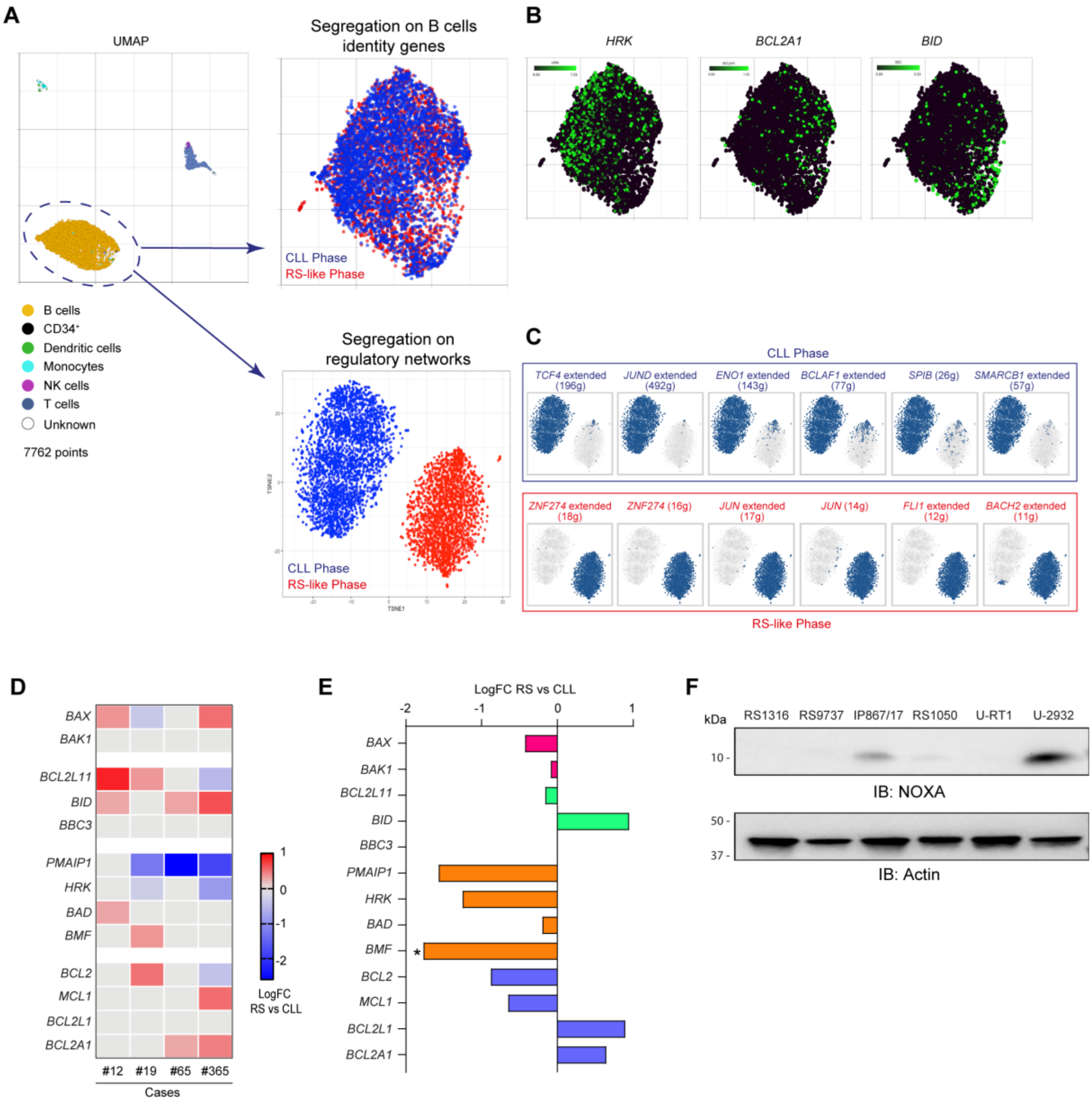

Supplemental figure 4

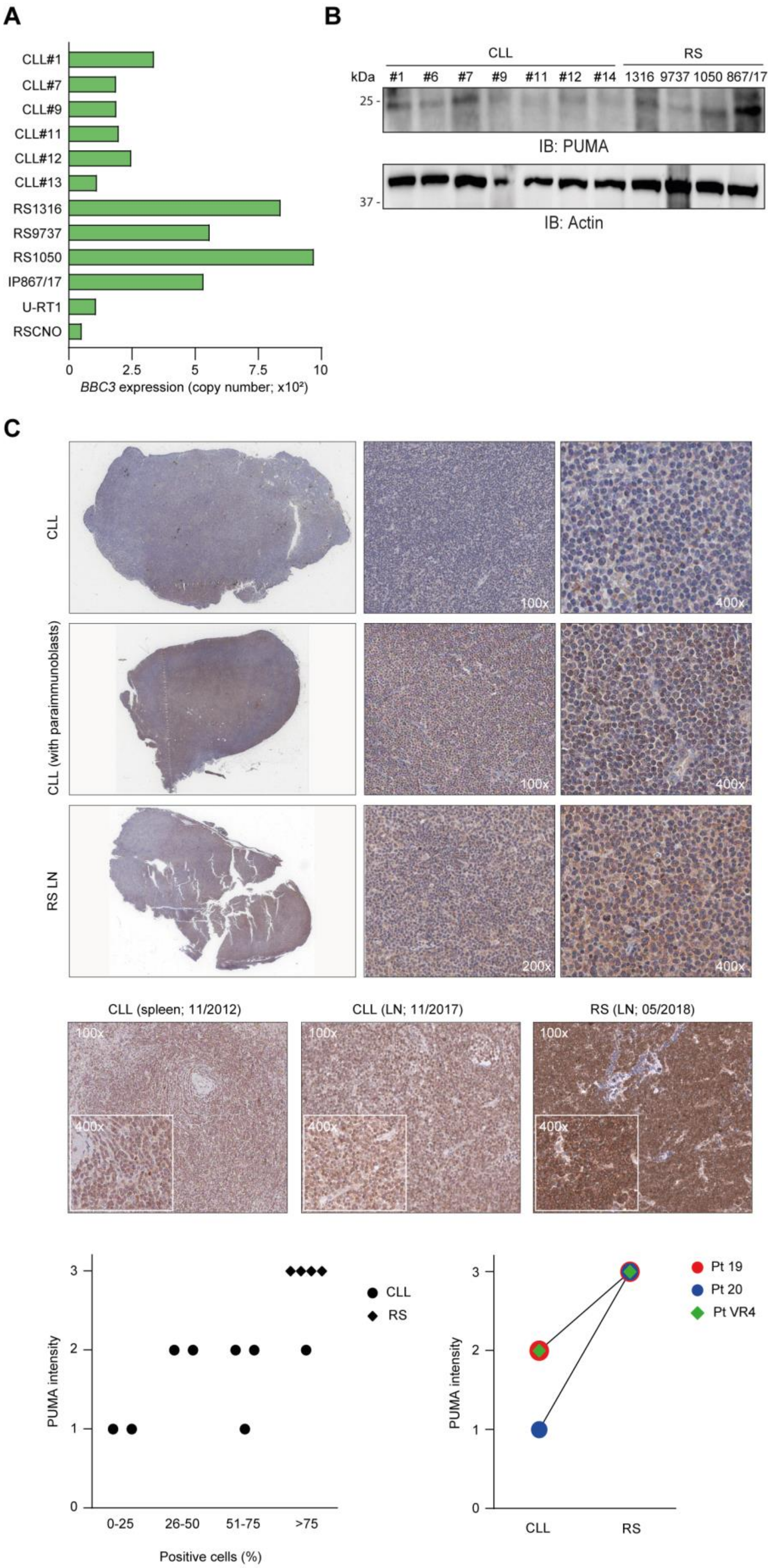

Supplemental figure 5

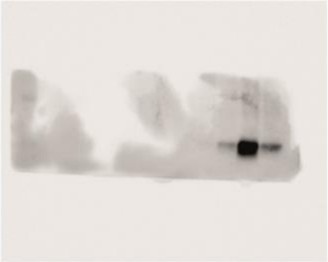

Immunoblot Bfl-1 (Figure 3D)

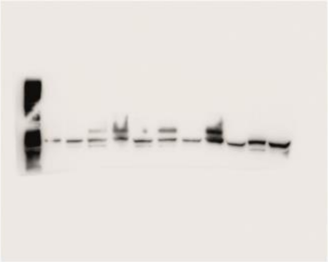

Immunoblot Clpb (Figure 7C)

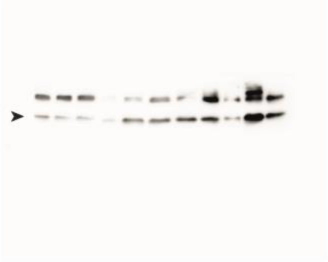

Immunoblot BID (Figure 6A)

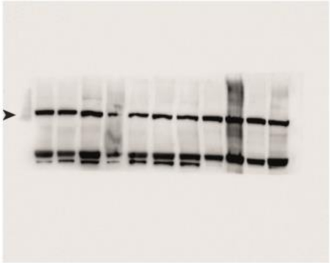

Immunoblot Actin (Figure 2D/S5)

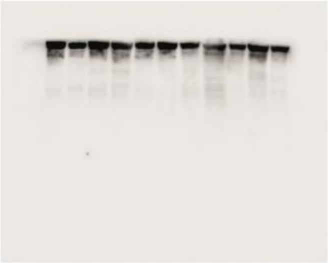

Immunoblot Actin (Figure 3D/6A/7C)

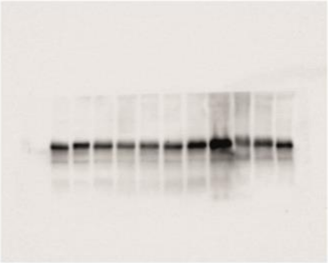

Immunoblot Bak (Figure 6A)

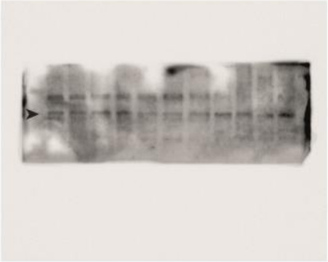

Immunoblot Bax (Figure 6A)

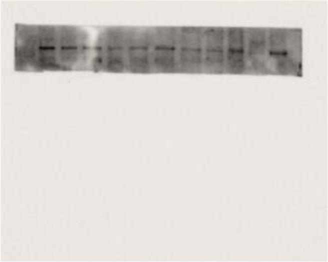

Immunoblot Bcl-2 (Figure 2D)

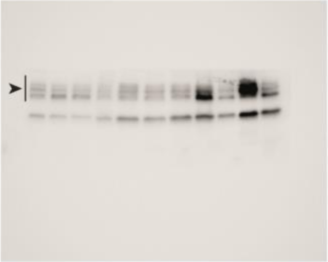

Immunoblot Bcl-xL (Figure 3D)

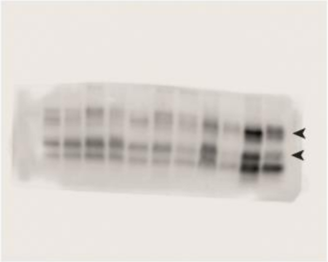

Immunoblot Bim (Figure 6A)

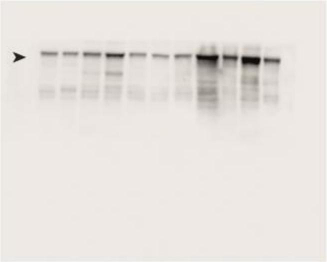

Immunoblot Mcl-1 (Figure 3D)

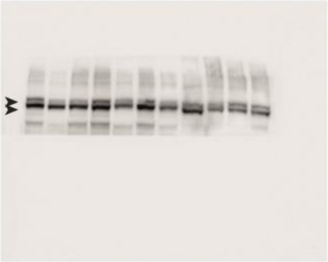

Immunoblot OPA1 (Figure 7C)

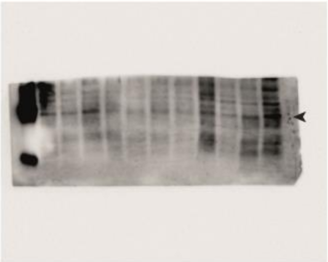

Immunoblot PUMA (Figure S5)

Supplemental figure 6
